# Differential expression of circulating miRNAs in maternal plasma in pregnancies with fetal macrosomia

**DOI:** 10.3892/ijmm.2014.1989

**Published:** 2014-10-31

**Authors:** QINYU GE, YANAN ZHU, HAILING LI, FEI TIAN, XUEYING XIE, YUNFEI BAI

**Affiliations:** 1 Key Laboratory for Child Development and Learning Science, Ministry of Education, Research Center for Learning Science, Southeast University, Nanjing 2100096, P.R. China; 2 State Key Laboratory of Bioelectronics, Southeast University, Nanjing 2100096, P.R. China; 3 Department of Gynecology and Obstetrics, Zhongda Hospital, Southeast University, Nanjing 210009, P.R. China

**Keywords:** circulating microRNAs, low-density array, fetal macrosomia, abnormal pregnancy

## Abstract

Macrosomia is associated with problems at birth and has life-long health implications for the infant. The aim of this study was to profile the plasma microRNAs (miRNAs or miRs) and evaluate the potential of circulating miRNAs to predict fetal macrosomia. The expression levels of miRNAs in plasma samples obtained from pregnant women with fetal macrosomia and from women with normal pregnancies (controls) were analyzed using TaqMan Low-Density Arrays (TLDAs) followed by quantitative reverse transcription polymerase chain reaction (RT-qPCR) validation and analysis. The TLDA data revealed that 143 miRNAs were differentially expressed in the plasma samples from pregnant women with fetal macrosomia compared with the controls (43 upregulated and 100 downregulated miRNAs). Twelve of these miRNAs were selected for RT-qPCR analysis. Receiver operational characteristic (ROC) curve analysis indicated that several miRNAs (e.g., miR-141-3p and miR-200c-3p) were clearly distinguished between pregnancies with fetal macrosomia and other types of abnormal pregnancy and healthy pregnancies with high sensitivity and specificity (AUC >0.9). The expression of miRNA clusters also showed a similar trend in pregnancies with fetal macrosomia. This study provides a platform for profiling circulating miRNAs in maternal plasma. Our data also suggest that altered levels of maternal plasma miRNAs have great potential to serve as non-invasive biomarkers and as a mechanistic indicator of abnormal pregnancies.

## Introduction

Pregnancies with a macrosomic fetus comprise a subgroup of high-risk pregnancies, which have been variously defined as pregancies in which the birth weight of the newborn is greater than 4,000, 4,500 or 5,000 g, while this fails to take gestational age and gender into consideration (1,2). Another definition of macrosomia as a birth weight >90th percentile for gestation or >97.75th percentile of a reference population corrected for gestational age and gender has been proposed. However, in light of the substantially increased adverse birth outcomes associated with a birth weight > 4,500 g, as well as its ease of use, this definition has been widely adopted (3,4). Maternal obesity, impaired glucose tolerance and gestational diabetes contribute to infant macrosomia; furthermore, the risk is higher in aboriginal populations (5–7). Macrosomia affects 50% of pregnancies complicated by maternal diabetes (8,9). It is also associated with problems at birth and has life-long health implications for the infant, including an increased risk of developing cardiovascular disease and diabetes in adulthood (10–12). Although the incidence of macrosomia is high amongst women with diabetes, there is currently no reliable method to predict who will deliver macrosomic infants. Understanding the factors that affect this placental growth is essential for the development of novel therapies. The study of circulating nucleic acid in maternal plasma may provide solutions for determining the development of macrosomia; for example, fetal macrosomia in pregnancies complicated by diabetes has been shown to accompany an increase in the levels of fetal deoxyribonucleic acid (DNA) and proteins in animal model systems (13).

MicroRNAs (miRNAs or miRs) are newly identified molecules that are common 18–25 nucleotide-long, non-coding RNA molecules that post-transcriptionally regulate gene expression by base-pairing with the 3′ untranslated region of complementary messenger RNA targets. miRNAs have been identified in animal cells and have been reported to play a role in numerous vital processes, including embryonic development, cellular differentiation and proliferation and apoptosis (14,15). They are produced in cells and tissues and have also been detected in the circulation recently. The aberrant expression of miRNAs has been associated with disease development and the recent discovery of miRNAs in serum and plasma has stimulated interest in their potential for use as circulating biomarkers (16). There is an increasing number of publications describing circulating miRNA biomarkers of breast, colon, gastric, lung, oral, ovarian, pancreatic, prostate, tongue and squamous cell cancers (17), stroke and neurological disorders (18), diabetes, heart failure and lupus erythematosus (19,20). Plasma/serum miRNA biomarkers have the advantage of stabilization and relative abundance, and their small size and consistent structure enables the application of quantitative multiplex assays using quantitative polymerase chain reaction (qPCR).

Circulating miRNA profiles or ‘signatures’ are altered in several disease states; thus, they are effective tools for the diagnosis and prognosis of diseases, including cardiovascular disease (21) and different forms of cancer (22–24). Establishing the plasma miRNA signature of pregnant women may therefore provide a non-invasive method for predicting fetal macrosomia.

In this study, we used TaqMan Low-Density Arrays (TLDAs) and reverse transcription qPCR (RT-qPCR) to profile the plasma miRNAs differentially expressed in pregnant women with fetal macrosomia and women with normal pregnancies as the controls, and explored the potency of plasma miRNA expression profiles as an early diagnostic biomarker for fetal macrosomia.

## Materials and methods

### Sample collection and processing

Peripheral blood samples from 45 pregnant women collected during their second trimester (18–28 weeks of gestational age) were selected; the neonates of these women were later diagnosed with macrosomia at delivery. Another 30 samples from women with normal healthy pregnancies were collected during the same trimester as the controls. Furthermore, an additional 16 samples from pregnant women with preeclampsia at their second gestational ages (22–28 weeks) were also collected for differential diagnosis by qPCR (some of them were later diagnosed with preeclampsia). All the samples were recruited from Zhongda Hospital, Southeast University (Nanjing, China) and Wuxi Maternity and Child Health Care Hospital (Wuxi, China) between November 2011 and July 2013. Among the collected samples, 10 samples from pregnant women with fetal macrosomia and 10 samples from women with normal pregnancies (control) were selected and pooled as samples for use in TLDAs. Samples from 35 pregnant women with fetal macrosomia and from 20 women with normal pregnancies were used in RT-qPCR for validation of the array data. Although there is no consensus on the definition of fetal macrosomia, in light of the substantially increased adverse birth outcomes associated with a birth weight >4,500 g, as well as its ease of use, this definition has been widely adopted (4). In the present study, the diagnosis of macrosomia was in accordance with this standard. This study was approved by the Ethics Committee of Zhongda Hospital, Southeast University and written informed consent was obtained from all the pregnant women prior to participation.

EDTA blood was centrifuged at 1,600 × g for 10 min at 4°C, and plasma was transferred to new tubes followed by further centrifugation at 16,000 × g for 10 min at 4°C. The separated plasma samples were subjected to RNA isolation or placed in a −80°C freezer for short-term storage. Two plasma pools of the macrosomia and control groups were created by combining 10 samples (50 μl of sample) and mixing by inversion and 500 μl of each of these pools were used to extract RNA for TLDAs.

### RNA extraction

Total RNA containing small RNA was extracted from 500 ml of plasma using TRIzol^®^ LS Reagent (Invitrogen, Carlsbad, CA, USA) and the miRNeasy Mini kit (Qiagen, Hilden, Germany) with some modifications according to Ng’s report (25). Briefly, TRIzol LS Reagent was added to the plasma samples in volumetric ratios as previously described (26). Following phase separation by the addition of chloroform and centrifugation, 1.5 vol of 100% ethanol was added to the aqueous phase, and the mixture was loaded onto the miRNeasy column (Qiagen) according to the manufacturer’s instructions. The final elution volume was 30 μl. The concentrations of all RNA samples were quantified using the Qubit^®^ RNA HS Assay kit with the Qubit 2.0 fluorometer (Life Technologies, Grand Island, NY, USA) according to the manufacturer’s instructions. The concentration of the RNA extracted from the plasma ranged from 3.68 to 53.84 ng/ml.

### miRNA profiling using TLDAs

The ABI TaqMan miRNA Low-Density Arrays (Applied Biosystems, Foster City, CA, USA) were selected as the platform for global miRNA expression profiling. Approximately 500 ng of total RNA was reverse transcribed into complementary DNA (cDNA) using the TaqMan microRNA reverse transcription kit and the miRNA Megaplex™ RT pool set A and B, a set of 2 pre-defined pools of up to 380 stem-loop RT primers per pool that enable the simultaneous synthesis of cDNA for mature miRNAs. The cDNA products were loaded onto TaqMan Human microRNA A + B Cards Set version 3.0, enabling the simultaneous quantification of 754 human miRNAs. TLDAs were performed on the ABI 7900HT Instrument (Applied Biosystems). Normalization was performed with the small nuclear RNAs (snRNAs) U44, U48 and U6. Quantitative miRNA expression data were acquired and normalized using ABI 7900HT SDS software (Applied Biosystems).

### RT-qPCR

RT-qPCR was used to detect and quantify individual miRNAs on real-time PCR instruments, ABI 7500 (Life Technologies). cDNA was synthesized from total RNA using specific stem-loop primers according to the miRNA reverse transcription protocol. The 20-μl PCR reaction mixture included 2 μl of RT product, 10 μl of SYBR^®^ Premix Ex Taq™ PCR master mix (2X concentration), 1 μl of the forward and reverse primers each and 6 μl of nuclease-free water. The primer sequences are presented in Table I. The reactions were carried out with a 10-min incubation at 95°C followed by 40 cycles of 95°C for 15 sec and 60°C for 1 min. All reactions were run in triplicate, and the average threshold cycle and SD values were calculated. The CT value is defined as the fractional cycle number at which the fluorescence exceeds the defined threshold. The data were analyzed with automatic settings for assigning the baseline. U6 snRNA levels were used as an internal normalization control and the expression level of the miRNAs was calculated using the ΔΔCT method.

### Statistical analysis

For RT-qPCR data, the relative expression levels of each target miRNA (Log2 relative level) were calculated according to the difference in CT values between the target miRNAs and U6 snRNA (ΔCT). CT values <35 were indicative of expression. Statistical analysis was performed using SPSS software version 16.0 (SPSS, Inc., Chicago, IL, USA) and GraphPad Prism 5 (GraphPad Software Inc., La Jolla, CA, USA). A P-value <0.05 was considered to indicate a statistically significant difference (Student’s t-test). For individual differentially expressed miRNAs, a receiver operating characteristic (ROC) curve was generated. The area under the curve (AUC) and 95% confidence intervals (CI) were calculated to determine the specificity and sensitivity of the miRNAs to predict fetal macrosomia.

## Results

### Demographics

The maternal characteristics are shown in Table II. The maternal age and pre-gestational body mass index (BMI) of the macrosomia group were higher than of those of the control group, and some women had a family history of hypertension and/or previous abortion; these data were consistent with those previous reported (27). No significant differences in gestational weight gain (GWG) were found between the pregnant women with fetal macrosomia and the women with normal pregnancies (controls) in this study. The maternal characteristics were consistent between the samples used for TLDAs and RT-qPCR.

### miRNA expression profiling of plasma in pregnancies with fetal macrosomia by TLDA

**Figure 1:**
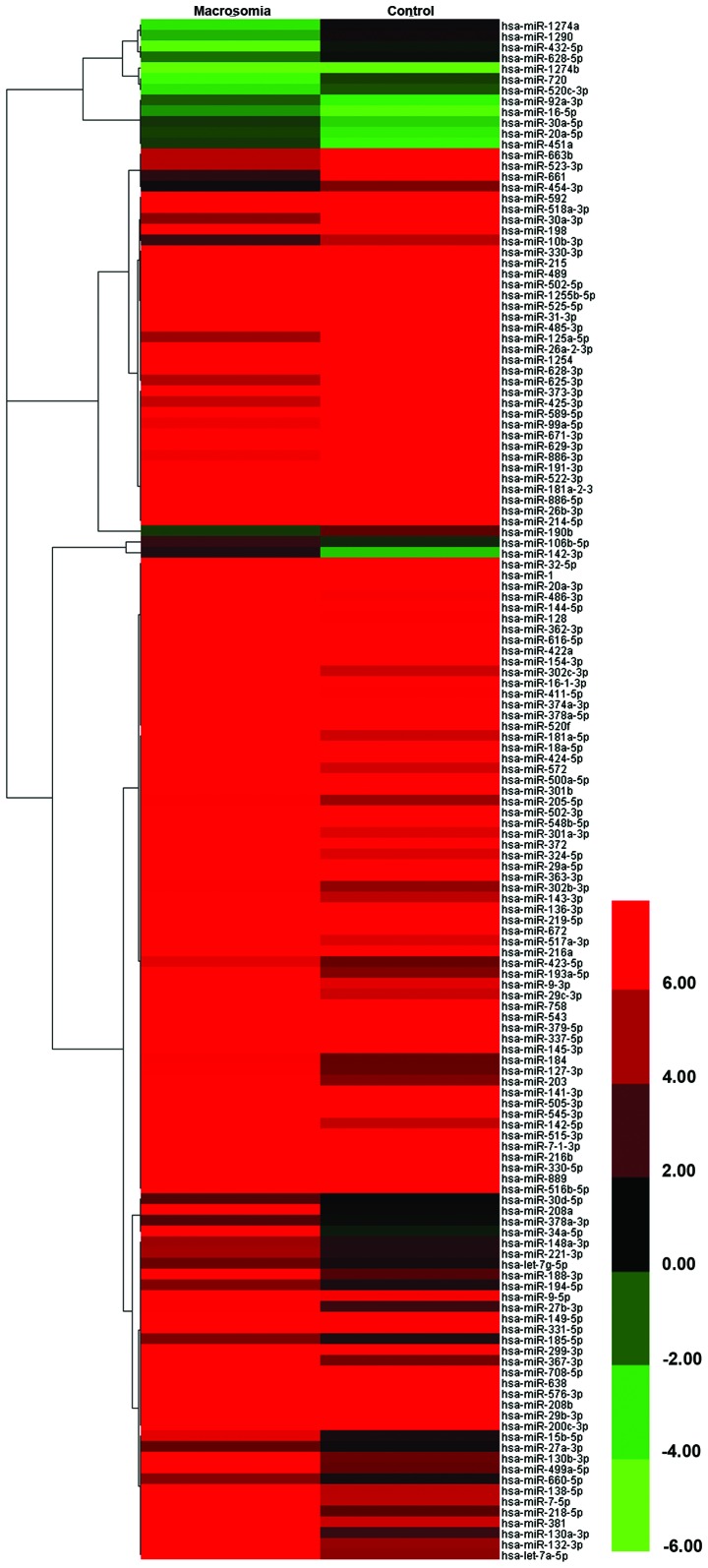
Heatmap of differential microRNA (miRNA) expression in plasma from pregnant women with fetal macrosomia and normal controls. The expression profiles of all the miRNAs that were assayed by TaqMan Low-Density Array (TLDA) are shown. The column shows fetal macrosomia and control groups normalized to endogenous controls, and the line shows the fold change in expression of each miRNA in each group. The color of each pattern indicates the fold change as Log2 (2−ΔCt) from green to red as from −8 to 8.

TaqMan Human miRNA Low-Density Array analysis was performed to identify circulating miRNAs exhibiting altered levels in pregnant women with fetal macrosomia, and plasma miRNAs were compared with the normal pregnancy controls. A total of 274 miRNAs incorporated in the array were detected; among these, 196 miRNAs were co-expressed; 223 and 248 miRNAs were detected in the plasma in pregnancies with fetal macrosomia and the healthy controls, respectively. To identify macrosomia-specific candidate miRNAs, the differential expression of miRNAs between the 2 groups was required to meet 2 criteria: i) CT values <35 to enable reliable detection; and ii) miRNA levels exhibiting ≥4-fold difference (ΔΔCT ≥2 or ΔΔCT ≤-2) between the macrosomia and control groups. A total of 143 miRNAs met these criteria, 43 of which were upregulated and 100 were downregulated in the pregnant women with fetal macrosomia compared with the normal pregnancy controls (Fig. 1). The top 20 differentially expressed miRNAs are listed in Table III.

Among these differentially expressed miRNAs between the pregnant women with fetal macrosomia and the normal pregnancy controls, 12 miRNAs were selected for further analysis according to TLDA results and the reults of previous reports studies (28–30). Four selected miRNAs (miR-661, miR-523-3p, miR-125a-5p and miR-30a-3p) were upregulated and 8 miRNAs were downregulated (miR-181a-5p, miR-200c-3p, miR-143-3p, miR-221-3p, miR-16-5p, miR-141-3p, miR-18a-5p and miR-451a), which contained not only significantly differentially expressed miRNAs, but also moderately differentially expressed miRNAs. Among these, miR-661, miR-523-3p, miR-200c-3p and miR-141-3p were significantly differentially expressed, while the other miRNAs were moderately differentially expressed; the majority of these miRNAs are related to obesity (miR-143-3p, miR-221-3p, miR-125a-5p, miR-16-5p (28-30) and cancer (miR-141-3p, miR-181a-5p, miR-18a-5p, miR-30a-3p) (31). miR-451a was highly abundant in red blood cells. It is also the most abundant miRNA in the sequencing data of our previously published study on abormal pregnacy (32).

**Figure 2:**
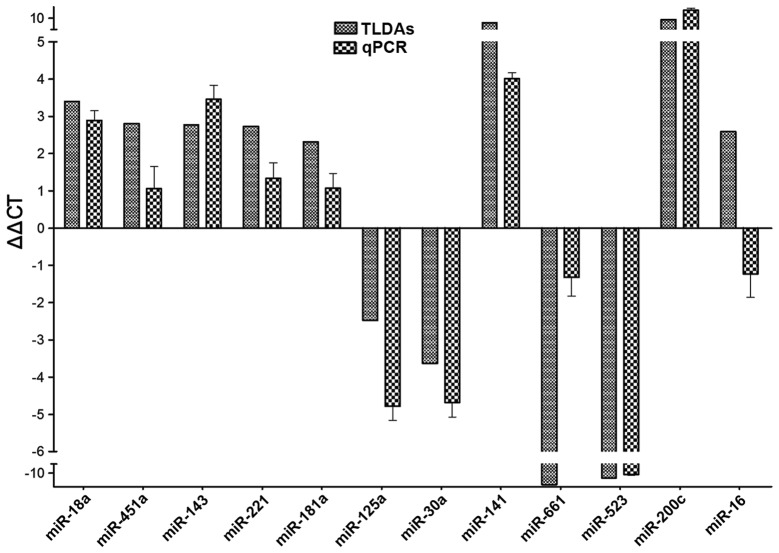
MicroRNA (miRNA) expression levels of validation of TaqMan Low-Density Arrays (TLDAs) and quantitative reverse transcription polymerase chain reaction (RT-qPCR). The left bar of each miRNA represents the results of TLDAs comparing fetal macrosomia with normal pregnancy (ΔΔCT); the right bar of each miRNA represents the resultsof RT-qPCR comparing fetal macrosomia with normal pregnancy (ΔΔCT).

Fig. 2 shows the qPCR confirmation results, which indicated that the differential expression obtained in the majority of the selected miRNAs between pregnant women with fetal macrosomia and the normal controls were consistent. The results confirmed the upregulated miRNAs, miR-523-3p, miR-125a-5p, miR-30a-3p and miR-661; and miR-221-3p, miR-451a, miR-143-3p, miR-18a-5p, miR-141-3p, miR-181a-5p and miR-200c-3p were confirmed as downregulated miRNAs in the plasma from pregnant women with macrosomia, while opposite results were obtained by RT-qPCR as regards miR-16-5p when compared with the TLDA results.

### RT-qPCR of miRNA expression in plasma from pregnant women with fetal macrosomia

**Figure 3:**
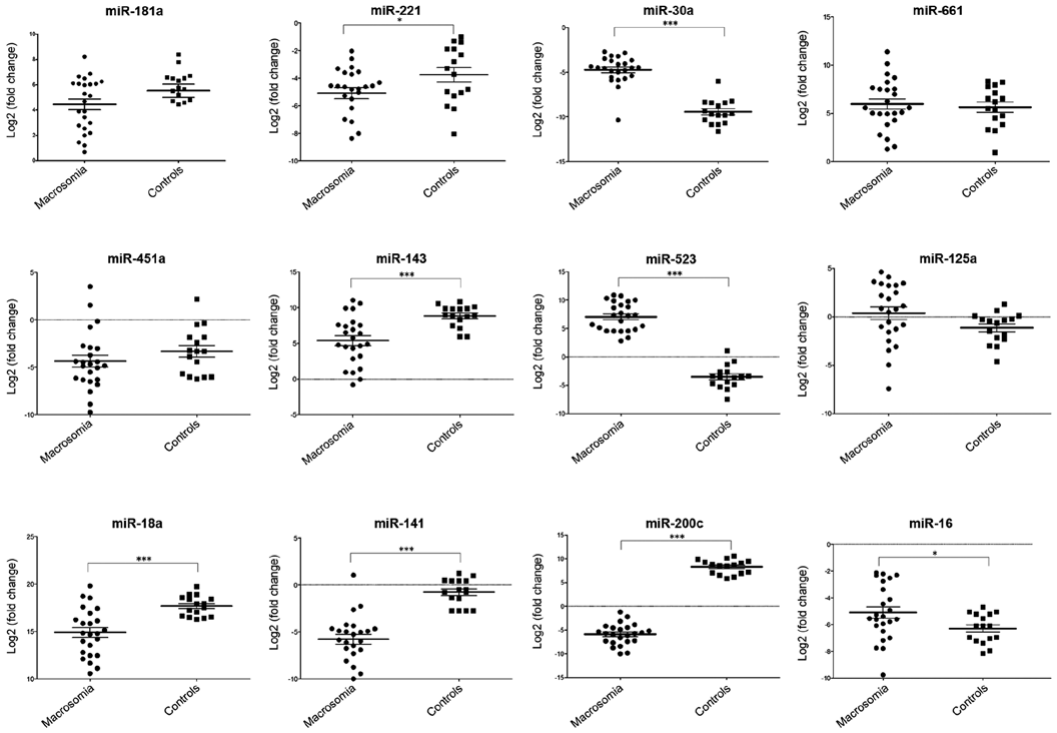
Plasma microRNAs (miRNAs) selected for verification by quantitative polymerase chain reaction (qPCR) in individual pregnant women carrying fetuses with macrosomia and normal pregnancy controls. The levels of plasma miR-221-3p, miR-143-3p, miR-18a, miR-141 and miR-200c were significantly lower in the pregnant women with fetal macrosomia compared with the normal controls (^***^P<0.001, ^*^P<0.05); miR30a, miR-523-3p and miR-16 were significantly higher in the pregnant women with fetal macrosomia, while no significant differences were detected in the expression of miR-181a, miR-661, miR125a and miR-451a. The expression levels of the miRNAs were normalized to U6 snRNA [Log2 (fold change)].

The majority of the miRNAs selected for verification were confirmed and quantified by RT-qPCR. Although no reliable endogenous control miRNA has been identified in studying circulating miRNAs (33,34), U6 snRNA is still the most common choice. Therefore, we used U6 snRNA as the endogenous control in this study and the expression levels of the miRNAs were normalized to U6 snRNA [Log2 (fold change)]. The expression levels of miR-221-3p, miR-143-3p, miR-18a-5p, miR-141-3p and miR-200c-3p were significantly lower in the plasma from women with fetal macrosomia compared with the normal controls (P<0.001, P<0.05, t-test). The levels of miR-523-3p, miR-30a-3p and miR-16-5p were significantly higher in the plasma from pregnant women with fetal macrosomia, while no significant differences were detected in the expression levels of miR-181a-5p, miR-661, miR125a and miR-451a. The results are shown in Fig. 3.

### Evaluation of the diagnostic potential of miRNAs for fetal macrosomia

**Figure 4:**
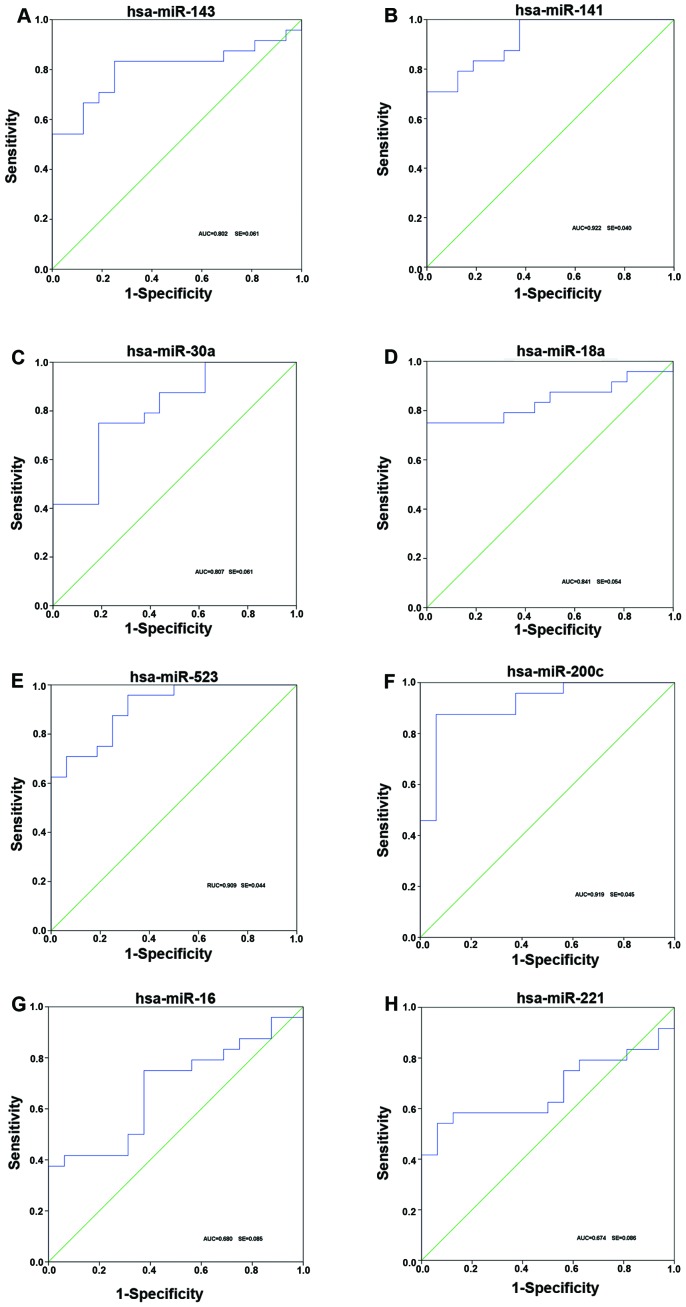
Receiver operating characteristic (ROC) curves of differentially expressed microRNAs (miRNAs) in pregnant women with fetal macrosomia and normal pregnancy controls. (A–H) ROC curves of miR-143-3p, miR-141, miR-30a, miR-18a, miR-523-3p, miR-200c, miR-221-3p and miR-16. miR-141, miR-523-3p and miR-200c showed a higher distinguishing efficiency, whereas the other miRNAs showed a moderate distinguishing efficiency.

To investigate the characteristics of these miRNAs as potential diagnostic biomarkers of fetal macrosomia, ROC curve analysis was performed on the miRNAs exhibiting significant differences in expression. The ROC curves of miR-30a-3p, miR-143-3p and miR-18a-5p exhibited a moderate distinguishing efficiency with an AUC value of 0.807 (95% CI), 0.802 (95% CI) and 0.841 (95% CI), respectively (Fig. 4A, C and D). miR-221-5p and miR-16-5p showed a relatively lower distinguishing efficiency with an AUC value of 0.680 (95% CI) and 0.674 (95% CI) (Fig. 4G and H), while miR-523-3p, miR200c and miR141 exhibited a higher distinguishing efficiency with an AUC value of 0.909 (95% CI), 0.948 (95% CI) and 0.940 (95% CI), respectively, which reflected a marked difference between the pregnant women with fetal macrosomia and those with normal pregnancy (controls) (Fig. 4B, E and F).

**Figure 5:**
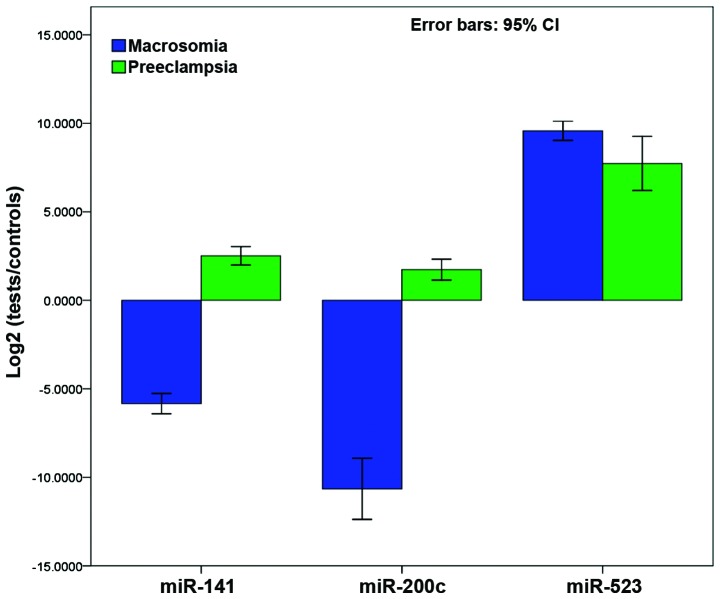
Comparison of the higher distinguishing efficiency microRNAs (miRNAs) between pregnant women with fetal macrosomia and those with other abnormal pregnancies. All 3 miRNAs detected were upregulated in the women with pregnancies complicated by preeclampsia, while miR-141 and miR-200c were downregulated and miR-523-3p was upregulated in the pregnant women with fetal macrosomia.

To verify the specificity of the miRNAs for fetal macrosomia, the 3 miRNAs with higher distinguishing efficiency from the plasma pools of abnormal pregnancy due to preeclampsia were also detected. Fig. 5 shows the results of the comparison between pregnant women with fetal macrosomia and pregnant women with preeclampsia. More specifically, miR-141-3p and miR-200c-3p showed significant differences, whereas no significant differences were observed for miR-523-3p (P=0.076). All 3 miRNAs were found to be upregulated in the pregnant women with preeclampsia, while miR-141-3p and miR-200c-3p were downregulated in the pregnant women with fetal macrosomia.

**Figure 6:**
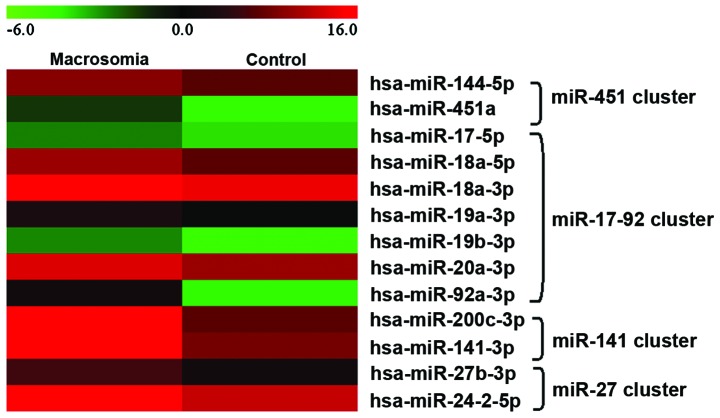
Expression levels of cluster members in pregnant women with fetal macrosomia and normal controls. miR-141, miR-451a, miR-17-92 and miR-27 clusters are shown. The same changing trend was observed in the expression levels of the cluster members in the maternal plasma from pregnant women with fetal macrosomia.

Four miRNA clusters were found to be differentially expressed in the maternal plasma of pregnant women with fetal macrosomia. A comparison of their expression levels indicated that the expression levels of the cluster members changed with a similar trend (Fig. 6). The results of ROC curve analysis revealed that the combination of the miRNA cluster members showed greater sensitivity and specificity. This suggested that the combination of the miRNA cluster members showed greater distinguishing efficiency for the diagnosis of fetal macrosomia. Furthermore, ROC curve analysis showed greater distinguishing efficiency when the miRNAs were combined with BMI and GWG (data not shown).

### Effect of pre-pregnancy BMI and GWG on fetal macrosomia

In this study, the effects of BMI and GWG on fetal macrosomia were also investigated. BMI is a measure of relative weight based on an individual’s mass and height, and has been reported to be related to obesity, diabetes mellitus, etc. The question addressed in the present study was whether a woman’s aberrant BMI level is associated with a greater risk of delivering an overweight newborn. Significant birth weight differences were found between the pregnant women with different BMIs (P<0.001), particularly those with a BMI >25 or BMI <18. GWG is another relevant factor that may be associated with fetal macrosomia. In this study, the results revealed that pregnant women with a GWG <10 or a GWG >20 were at a greater risk of carrying a macrosomic fetus (data not shown).

## Discussion

In the present study, circulating miRNAs in maternal plasma in pregnancies with fetal macrosomia were detected and analyzed. Although miRNAs have been extensively investigated as novel and non-invasive diagnostic and prognostic markers in various types of cancer and abnormal pregnancies (32,35–37), to the best of our knowledge, this is the first comprehensive survey on maternal circulating miRNA expression profiles in maternal plasma from pregnant women with fetal macrosomia. We investigated circulating miRNAs in the maternal plasma from pregnant women with fetal macrosomia by TLDAs. Differentially expressed miRNAs were profiled, and some of them were validated by RT-qPCR.

Although the concentration of circulating miRNAs in maternal plasma is rare, 274 types of miRNAs were detected with TLDA. Among these, a total of 196 miRNAs were co-expressed; 27 and 52 miRNAs were detected only in the plasma of carriers of macrosomic fetuses and healthy controls, respectively. Forty additional individual plasma samples were detected by RT-qPCR as validation since only 2 pooled samples (each included 10 individual samples) were measured by TLDA. Twelve miRNAs were selected for RT-qPCR based on the TLDA results; consistent results were obtained for all of the miRNAs apart from miR-16-5p (Fig. 1). This inconformity may be mainly due to the individual differences of the investigated plasma samples.

To evaluate the efficiency of these selected dysregulated miRNAs for the diagnosis of fetal macrosomia, ROC curves were constructed for each miRNA. miR-141-3p, miR-523-3p, and miR-200c-3p showed the ability to distinguish fetal macrosomia from normal control pregnancy efficiently, with an AUC value >0.9. In addition, miR-141-3p and miR-200c-3p showed the ability to distinguish fetal macrosomia from other types of abnormal pregnancy, such as preeclampsia. These miRNAs may thus be potential biomarkers for the detection and diagnosis of fetal macrosomia and other complications associated with pregnancy. However, the biological mechanisms of the dysregulation of these miRNAs and their therapeutic potential in fetal macrosomia require further investigation.

We also analyzed the expression of miRNA clusters. Of note, the results revealed that cluster members showed similar changes in expression in pregnancy (Fig. 6). miRNA clusters are largely present in metazoan genomes with the diversity of their distribution (38). A number of miRNAs are linked as clusters on chromosomes and are often transcribed from genomic DNAs as a single polycistronic transcript to provide the opportunity for different miRNAs to target several categories of genes simultaneously (39,40). Accumulating evidence suggests that clustered miRNAs are always transcribed as polycistrons and have similar expression patterns (41). In this study, we observed the characteristics of miRNA cluster expression patterns. Four miRNA clusters, the miR-451, miR-17-92, miR-27 and miR-141 clusters were found to be significantly differentially expressed in the plasma from pregnant women with fetal macrosomia. Although their expression levels differed, the results revealed that the changes in the expession of the cluster members showed a similar trend when compared to the expression patterns.

Maternal obesity, impaired glucose tolerance, gestational diabetes and high BMI may all contribute to infant macrosomia (6,9). In this study, we detected the miRNA expression and analyzed the correlation between BMI and miRNA expression levels. miRNAs, such as miR-143-3p and miR-221-3p have been reported to be related to obesity and diabetes (28–31), as miRNA-143 inhibits insulin-stimulated AKT activation and impairs glucose metabolism. These miRNAs were also found to be significantly differentially expressed in the plasma of pregnant women with fetal macrosomia in this study, while opposite trends in expression were observed in our study. For instance, miR-143-3p has shown an upregulated expression in obesity (30), while its expression was downregulated in the pregnant women with fetal macrosomia in our study. We hypothesized that miRNAs may show a compensatory reverse expression in childhood and in adulthood compared with the embryonic phase.

GWG is also greatly considered a factor that may be related to abnormal pregnancy. Although no significant differences in GWG were observed between the macrosomia and the control groups in this study, surprisingly, it was found that infants with the highest birth weight were delivered by women with the lowest GWG. Therefore, the association between GWG, miRNAs and macrosomia requires further investigation.

The differentially expressed miRNAs which were detected by TLDA with a ΔΔCT value >2.0 or < −2.0 in pregnant women with fetal macrosomia compared with the normal pregnancy controls, were regarded as macrosomia-specific plasma miRNAs. We collected their experimentally validated target sites from the miRTarBase database (42). Those genes targeted by >2 miRNAs were regarded as target genes of maternal plasma miRNAs. We analyzed functions and functional relationships of these target genes using Gene Ontology Analysis and Kyoto Encyclopedia of Genes and Genomes (KEGG). The results revealed that genetic disorders, immunological disease, cell signaling, cancer and cell cycle were the enriched pathways of maternal plasma miRNA target genes (data not shown). These pathways are closely related to pregnancy progression. Therefore, maternal plasma miRNAs may participate in the regulation of pregnancy through these pathways.

This study had several limitations which should be noted. Firstly, only partially dysregulated miRNAs were selected and evaluated, while other differentially expressed miRNAs were not detected in this study. More efficient biomarkers and significant miRNAs may still be detected for research on fetal macrosomia. Secondly, our study is a preliminary investigation of miRNA expression levels associated with abnormal pregnancy, fetal macrosomia in particular, and provides a platform for related research. More information is required on biomarkers, including exact differences in individual pregnancies, as well as in situations of other types of abnormal pregnancy. More importantly, the functions and miRNA regulatory pathways should be thoroughly investigated. Furthermore, additional research conducted on larger numbers of pregnant women (abnormal pregnancies and normal controls) and non-pregnant healthy controls is required to validate our findings.

In conclusion, TLDAs revealed the dysregulation of 143 miRNAs in the maternal plasma from pregnant women with fetal macrosomia. miR-141-3p and miR-200c-3p were shown to be able to distinguish pregnancies with fetal macrosomia from healthy pregnancies and other types of abnormal pregnancy with high sensitivity and specificity. Strong correlations were observed between miRNA expression levels and BMI. It was also revealed that the expression of miRNA clusters showed the same changing trend in fetal macrosomia. This suggests that altered levels of miRNAs in maternal plasma have great potential to serve as non-invasive and mechanistically relevant biomarkers for the detection and diagnosis of abnormal pregnancies.

## Figures and Tables

**Table I:** DNA sequences of the primers used for RT-qPCR.

Name	DNA sequence (5′→3′)
miR-451a-RT primer	GTCGTATCCAGTGCAGGGTCCGAGGTATTCGCACTGGATACGACAACTCA
miR-451a-Forward primer	GAAACCGTTACCATTACTGAG
miR-16-5p-RT primer	GTCGTATCCAGTGCAGGGTCCGAGGTATTCGCACTGGATACGACCGCCAA
miR-16-5p-Forward primer	GTAGCAGCACGTAAATATTGGC
miR-181a-5p-RT primer	GTCGTATCCAGTGCAGGGTCCGAGGTATTCGCACTGGATACGACACTCAC
miR-181a-5p-Forward primer	GAACATTCAACGCTGTCGGTGA
miR-661-RT primer	GTCGTATCCAGTGCAGGGTCCGAGGTATTCGCACTGGATACGACACGCGC
miR-661-Forward primer	GTGCCTGGGTCTCTGGCCTGCG
miR-200c-3p-RT primer	GTCGTATCCAGTGCAGGGTCCGAGGTATTCGCACTGGATACGACTCCATC
miR-200c-3p-Forward primer	GTAATACTGCCGGGTAATGATG
miR-221-3p-RT primer	GTCGTATCCAGTGCAGGGTCCGAGGTATTCGCACTGGATACGACGAAACC
miR-221-3p-Forward primer	GAGCTACATTGTCTGCTGGGT
miR-143-3p-RT primer	GTCGTATCCAGTGCAGGGTCCGAGGTATTCGCACTGGATACGACGAGCTA
miR-143-3p-Forward primer	GTGAGATGAAGCACTGTAGC
miR-141-3p-RT primer	GTCGTATCCAGTGCAGGGTCCGAGGTATTCGCACTGGATACGACCCATCT
miR-141-3p-Forward primer	GTAACACTGTCTGGTAAAGATG
miR-18a-5p-RT primer	GTCGTATCCAGTGCAGGGTCCGAGGTATTCGCACTGGATACGACCTATCT
miR-18a-5p-Forward primer	GTAAGGTGCATCTAGTGCAGA
miR-523-3p-RT primer	GTCGTATCCAGTGCAGGGTCCGAGGTATTCGCACTGGATACGACACCCTC
miR-523-3p-Forward primer	GGAACGCGCTTCCCTATAGAGG
miR-125a-3p-RT primer	GTCGTATCCAGTGCAGGGTCCGAGGTATTCGCACTGGATACGACGGCTCC
miR-125a-3p-Forward primer	GACAGGTGAGGTTCTTGGGAG
miR-30a-3p-RT primer	GTCGTATCCAGTGCAGGGTCCGAGGTATTCGCACTGGATACGACGCTGCA
miR-30a-3p-Forward primer	GCTTTCAGTCGGATGTTTGCA
Universal Reverse primer	GTGCAGGGTCCGAGGT
U6-RT primer	AACGCTTCACGAATTTGCGT
U6-Forward primer	CTCGCTTCGGCAGCACA
U6-Reverse primer	AACGCTTCACGAATTTGCGT

**Table II:** Epidemiological characteristics of pregnant women with fetal macrosomia and healthy controls.

	TLDA			qPCR validation		
		
	Case	Control	P-value	Case	Control	P-value
Number of samples	10	10	-	35	20	-
Maternal age (years)	28.34±4.67	25.31±3.41	<0.001	28.53±5.18	25.27±3.82	<0.001
BMI (kg/m^2^)	22.64±2.38	19.58±2.32	<0.001	22.75±2.72	19.41±1.74	<0.001
Previous abortion (%)	0	0	-	1.5%	0	-
Nulliparous	90.0%	100%	-	82.7%	94.0%	-
Family history of hypertension and diabetes	19%	0	21%	0	-	
Birth weight (kg)	4.87±0.43	3.27±0.41	<0.001	4.70±0.40	3.22±0.32	<0.001
GWG (kg)	15.56±4.3	14.18±4.26	0.456	15.42±4.6	14.23±4.14	0.492

TLDA, TaqMan Low-Density Array; qPCR, quantitative polymerase chain reaction; BMI, body mass index; GWG, gestational weight gain.

**Table III:** Top 20 differentially expressed miRNAs between pregnant women with fetal macrosomia and normal controls.

Upregulated in case group compared with control	ΔΔCT	Downregulated in case group compared with control	ΔΔCT
hsa-miR-661	−15.1039	hsa-miR-34a-5p	17.00549
hsa-miR-663b	−12.3943	hsa-miR-208a	16.0467
hsa-miR-523-3p	−12.2455	hsa-miR-499a-5p	13.6248
hsa-miR-198	−9.74231	hsa-miR-130b-3p	13.47667
hsa-miR-592	−8.84758	hsa-let-7a-5p	12.7783
hsa-miR-518a-3p	−8.50963	hsa-miR-132-3p	12.6045
hsa-miR-330-3p	−7.40832	hsa-miR-138-5p	11.94246
hsa-miR-502-5p	−7.13427	hsa-miR-7-5p	11.74449
hsa-miR-215	−6.94627	hsa-miR-381	11.6779
hsa-miR-489	−6.88626	hsa-miR-9-5p	10.87772
hsa-miR-485-3p	−6.44884	hsa-miR-331-5p	10.59712
hsa-miR-525-5p	−6.27043	hsa-miR-149-5p	10.59156
hsa-miR-26a-2-3p	−6.12089	hsa-miR-299-3p	10.18141
hsa-miR-1254	−5.94441	hsa-miR-708-5p	9.921302
hsa-miR-373-3p	−5.61778	hsa-miR-200c-3p	9.433656
hsa-miR-1255b-5p	−5.3922	hsa-miR-29b-3p	9.103284
hsa-miR-432-5p	−5.14086	hsa-miR-208b	8.918743
hsa-miR-31-3p	−5.04041	hsa-miR-576-3p	8.858621
hsa-miR-589-5p	−4.83138	hsa-miR-638	8.628476
hsa-miR-1274a	−4.30028	hsa-miR-545-3p	8.504154

miRNAs, microRNAs.
